# ER-mediated control for abundance, quality, and signaling of transmembrane immune receptors in plants

**DOI:** 10.3389/fpls.2014.00065

**Published:** 2014-02-25

**Authors:** Nico Tintor, Yusuke Saijo

**Affiliations:** ^1^Department of Plant Microbe Interactions, Max Planck Institute for Plant Breeding ResearchCologne, Germany; ^2^Laboratory of Plant Immunity, Graduate School of Biological Sciences, Nara Institute of Science and TechnologyIkoma, Japan; ^3^Japan Science and Technology, Precursory Research for Embryonic Science and TechnologyKawaguchi, Japan

**Keywords:** ERQC, ER stress, MAMP/DAMP, immune receptor, LRR/LysM

## Abstract

Plants recognize a wide range of microbes with cell-surface and intracellular immune receptors. Transmembrane pattern recognition receptors (PRRs) initiate immune responses upon recognition of cognate ligands characteristic of microbes or aberrant cellular states, designated microbe-associated molecular patterns or danger-associated molecular patterns (DAMPs), respectively.**Pattern-triggered immunity provides a first line of defense that restricts the invasion and propagation of both adapted and non-adapted pathogens. Receptor kinases (RKs) and receptor-like proteins (RLPs) with an extracellular leucine-rich repeat or lysine-motif (LysM) domain are extensively used as PRRs. The correct folding of the extracellular domain of these receptors is under quality control (QC) in the endoplasmic reticulum (ER), which thus provides a critical step in plant immunity. Genetic and structural insight suggests that ERQC regulates not only the abundance and quality of transmembrane receptors but also affects signal sorting between multi-branched pathways downstream of the receptor. However, ERQC dysfunction can also positively stimulate plant immunity, possibly through cell death and DAMP signaling pathways.

## INTRODUCTION

Plants sense their encounters to microbes through immune receptors that monitor extracellular or intracellular spaces for pathogen-associated ligands (Jones and Dangl, 2006; Boller and Felix, 2009; Dodds and Rathjen, 2010). Cell-surface receptors involve not only PRRs that recognize microbe-associated molecular patterns (MAMPs) or danger-associated molecular patterns (DAMPs) to confer pattern-triggered immunity (PTI), but also the resistance (R) proteins that recognize pathogen effectors to confer effector-triggered immunity (ETI). Among the former, FLS2 and EFR recognize the bacterial MAMPs flagellin (flg22 epitope) and the elongation factor EF-Tu (elf18 epitope), respectively (Gomez-Gomez and Boller, 2000; Zipfel et al., 2006). Among the latter, the tomato LRR-RLPs Cf proteins and Ve1 and the rice LRR-RK XA21 confer immunity against *Cladosporium fulvum*, *Verticillium dahliae*,**and *Xanthomonas oryzae*, respectively (Song et al., 1995; Rivas and Thomas, 2005; de Jonge et al., 2012). Adapted pathogens can escape or overcome the defenses mounted by these extracellular receptors, with the aid of effectors. However, pathogen effectors acting within the host cells are directly or indirectly recognized by intracellular nucleotide-binding and LRR (NB-LRR) domain-containing R proteins, which leads to strong ETI activation that terminates pathogen growth.

The ER plays a central role in the biogenesis and intracellular distribution of transmembrane receptors according to their folding states and cellular demands. Hence, the ER homeostasis and prompt adaptation to ER stress are vital for plant life and health.

## TRANSMEMBRANE IMMUNE RECEPTORS AND REGULATORS IN PLANTS

In plants, the extracellular leucine-rich repeat (LRR) and lysine-motif (LysM) domains define two major structural modules that recur in the extracellular domain of PRRs. PRRs include the LRR-RKs FLS2, EFR, and PEPR1/2 (for the endogenous Pep peptides; Krol et al., 2010; Yamaguchi et al., 2010), the LRR-RLPs LeEIX1/2 (for fungal xylanase; Ron and Avni, 2004), the LysM-RK CERK1 (for fungal chitin; Miya et al., 2007; Wan et al., 2008), the LysM-RLPs CEBiP (for fungal chitin; Kaku et al., 2006) and LYM1 and LYM3 (for bacterial peptidoglycan; Willmann et al., 2011). PRR-associated RKs and/or RLPs have been also described. The LRR-RK BAK1 (and/or its related SERK members) associates with numerous LRR-RKs, including the PRRs FLS2, EFR, and PEPR1/2, and also the defense regulators BIR1 and SOBIR1/EVR (Chinchilla et al., 2007; Ryan et al., 2007; Gao et al., 2009; Postel et al., 2010; Schulze et al., 2010). The tomato SOBIR1 ortholog interacts with Cf-4 and Ve-1 and is required for their ETI functions (Liebrand et al., 2013b). SOBIR1/EVR also acts together with the LRR-RLP RLP30, apparently in PTI against *Sclerotinia sclerotiorum* (Zhang et al., 2013). CERK1 also serves LYM1 and LYM3 in peptidoglycan perception (Willmann et al., 2011). Given the BAK1-independence of CERK1-mediated chitin signaling (Heese et al., 2007; Shan et al., 2008; Gimenez-Ibanez et al., 2009), the LRR-PRRs and LysM-PRRs might act in separate receptor complexes. In sum, extensive engagement of transmembrane receptors and regulators represents a key principle in plant immunity.

## EVOLUTIONARILY CONSERVED ERQC PATHWAYS UNDERLIE PLANT-SPECIFIC SECRETORY PROCESSES

In eukaryotes, including plants, the biogenesis of transmembrane or secretory proteins occurs through the ER. Folding status of these proteins are monitored during their folding and maturation by a mechanism termed ERQC that ensures the delivery of properly folded proteins to their functional sites (Anelli and Sitia, 2008). This is essential in all eukaryotes tested (Kelleher and Gilmore, 2006). One pathway employs the Hsp70 family member BiP that acts in a multi-protein complex with the Hsp40 family members ERdJ and stromal cell-derived factor 2 (SDF2; Meunier et al., 2002). A second pathway relies on Asn (N)-glycosylation (Glc_3_Man_9_GlcNAc_2_ conjugation) on the nascent client proteins catalyzed by the oligosaccharyltransferase (OST) complex. An N-glycosylation inhibitor, tunicamycin, is widely used as an inducer of ER stress in plants (Koizumi et al., 1999). Subsequent folding of N-glycosylated proteins occurs through a pathway involving glucosidases I and II (GI and GII), the folding cycle via the ER chaperones calreticulin (CRT) and calnexin (CNX), and UDP-glucose:glycoprotein glucosyltransferase (UGGT) that are highly conserved in eukaryotes (Kelleher and Gilmore, 2006). However, plants exhibit better tolerance to single gene disruptions in the N-glycosylation pathway downstream of the OST-mediated step (see below). *N*-glycoproteomics studies comparing seven model organisms including *Arabidopsis* also revealed the existence of lineage-specific N-glycosylated proteomes in a much larger portion than previously thought (Zielinska et al., 2012). Over-representation of extracellular functions in lineage-specific *N*-glycoproteomes implies a role for N-glycosylation in the divergence of extracellular and/or secretory functions unique to the plant lineage.

## ERQC FOR PLANT IMMUNE RECEPTORS AND REGULATORS

Genetic tractability for ERQC components in plant models facilitates unraveling their roles in different branches of plant immunity. Genetic studies on *Arabidopsis* elf18-hyposensitive mutants led to the discovery for an ER N-glycosylation pathway involving the OST complex subunits STT3A and OST3/6, CRT3, UGGT, GII that is essential for EFR but not FLS2 biogenesis (Li et al., 2009; Lu et al., 2009; Nekrasov et al., 2009; Saijo et al., 2009; Haweker et al., 2010; Farid et al., 2013). Consistently, compared to FLS2, EFR is highly vulnerable toward chemical interference with the OST function or N-glycosylation site substitutions in the receptor LRR domain (Nekrasov et al., 2009; Saijo et al., 2009; Haweker et al., 2010; Sun et al., 2012). In addition, EFR biogenesis also specifically requires SDF2, ERdj3b, and BiP (Nekrasov et al., 2009). It remains elusive how these ERQC pathways are coordinated.

This N-glycosylation pathway is also required for SA-inducible but EFR-independent resistance (Saijo et al., 2009). Consistent with this, SA-induced resistance is reduced in the absence of BiPs or the OST subunit DAD1 (Wang et al., 2005). By contrast, DAD1 is dispensable for EFR accumulation and function (Haweker et al., 2010). In *Nicotiana benthamiana *that inherently lacks EFR,**CRT3a is also required for PTI to the oomycete pathogen *Phytophthora infestans* (Matsukawa et al., 2013). These findings imply the existence of another ERQC client receptor(s) than EFR mediating these immune responses.

Genetic studies with misfolded but signaling-competent alleles of the LRR-RK brassinosteroid receptor BRI1, designated *bri1-5 *and* bri1-9,* have also revealed a role for a common set of ERQC components, CRT3, UGGT, and BiP, in the ER retention of the BRI1 variants (Jin et al., 2007, 2009; Hong et al., 2008). However, OST3/6 seems to be dispensable for this ERQC (Farid et al., 2013). This again points to partial client-specific divergence in the usage of the OST complex subunits in the N-glycosylation pathway. The overall composition and precise mode of actions for the OST complex subunits remain to be elucidated.

Proteomics studies revealed BiPs and CRTs among Cf-4- and Ve1 interacting proteins *in vivo* (Liebrand et al., 2012, 2013a). Of four BiP members and three CRT members in tomato and *N. benthamiana*, only silencing of CRT3a (a plant-specific CRT; Christensen et al., 2010) compromises the proper glycosylation and biogenesis of functional Cf-4 protein. By contrast, silencing of single BiP members (except BiP4) and CRT members impaired Ve1-mediated resistance, without a significant decrease in the accumulation of complex glycan-conjugated Ve1. It is possible that these ER chaperones rather serve the LRR-RKs SOBIR1, SERK1, or BAK1 that is required for Ve1 function (Fradin et al., 2009, 2011; Liebrand et al., 2013b). Likewise, BiP3, SDF2, ERdj3B, CNX1, and CRT3 were recovered among XA21-associated proteins from rice plants (Park et al., 2010, 2013). BiP3 overexpression and SDF2 silencing both lower XA21-mediated resistance, pointing to a critical role for the ER homeostasis in this receptor pathway.

Compared to CRT3, CRT2 appears to have a minor role in PTI (Li et al., 2009; Christensen et al., 2010). True CRT2 function might be obscured by its dual role: CRT2 overexpression in *Arabidopsis* led to constitutive SA accumulation and *PR *gene activation in a manner dependent on its C-terminal Ca^2^^+^-binding domain, but it rather lowered bacterial resistance (Qiu et al., 2011). However, the perturbation of the N-terminal chaperone domain allowed CRT2 to enhance bacterial resistance. It might be that CRT2 chaperone function serves a defense suppressor whilst Ca^2^^+^-buffering function promotes SA-based immunity. In *N. benthamiana*, both CRT2 and CRT3 are required for the expression of the LRR-RK IRK1 that is essential for the NB-LRR R protein-mediated ETI to *Tobacco mosaic virus* (Caplan et al., 2009).

CRT-mediated defense suppression is exploited by the root-knot nematode *Meloidogyne incognita *for virulence promotion**(Jaouannet et al., 2013). The nematode secretes CRT, which can suppress MAMP responses, into the apoplastic spaces during plant infection. In plants and animals, CRTs localize not only inside but also outside the ER (Baluska et al., 1999; Sharma et al., 2004; Krysko et al., 2013). Whether extracellular CRT pools modulate immunity in plants, like in mammals, requires future investigation.

In contrast to the aforementioned receptors that strictly require a subset of ERQC components for their biogenesis and/or function, there are also receptors that exhibit relative robustness to ERQC dysfunction, such as FLS2 and PEPR1/2 (Tintor et al., 2013). Future studies will be required to elucidate the molecular determinants for the differences in ERQC dependency.

## ROLE FOR THE LRR DOMAIN CONFORMATION IN IMMUNE RECEPTOR COMPLEX FORMATION AND SIGNALING

**FIGURE 1:**
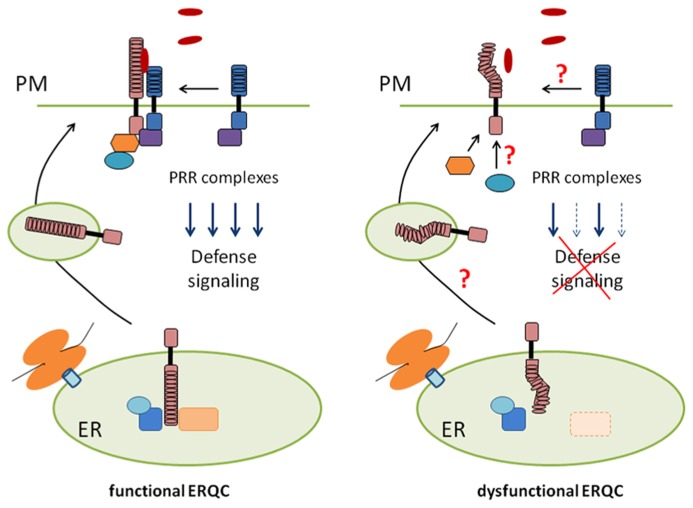
**A model for ER-mediated control of PRR biogenesis and signaling**. In weakly dysfunctional alleles of ERQC components (right), folding defects of the extracellular domain of PRRs might affect stable accumulation at the plasma membrane (PM), subcellular trafficking, assembly of pre- and post-recognition complexes, or combinations thereof. Importantly, this can selectively impair a subset of diverse signaling outputs downstream of the receptor.

The question of how a single receptor governs diverse signaling outputs represents an important challenge in receptor biology. In weakly dysfunctional alleles of CRT3 and GII, the tested signaling outputs of EFR are differentially, but not uniformly, impaired without a significant decrease in the receptor steady-state levels (Lu et al., 2009; Saijo et al., 2009). In these alleles, the degree of signaling defects is correlated with that of decreases in EFR-ligand binding. It remains elusive how the lowered ligand binding differentially affects multi-branched signaling pathways emanating from the receptor. Nevertheless, these findings predict that signal separation for these diverse outputs occurs at the level of or in the proximity to the receptor, and can be influenced by the folding states of the LRR domain (**Figure 1**).

A crystal structure of flg22-bound FLS2 and BAK1 ectodomains revealed that the FLS2 ectodomain forms a superhelical structure with the flg22 binding site in the concave surface of the LRR 3–16 (Sun et al., 2013). This is in agreement with previous studies on the receptor-ligand binding (Dunning et al., 2007; Mueller et al., 2012). Importantly, flg22 binding occurs in the FLS2-BAK1 interface and seems to stabilize their interaction. Strikingly, perturbations of the FLS2-BAK1 interface differentially affect separate signaling outputs. BAK1 point substitutions in key residues of this interface almost abolished MPK4/MPK11 activation without substantial effects on MPK3/MPK6 activation. Flg22 variants incapable of BAK1 binding reduce FLS2-BAK1 interaction and a ROS burst without affecting MPK activation. These results suggest that changes in either FLS2 or BAK1 LRR conformation alter the receptor complex formation and function, and importantly, which selectively influences downstream signaling pathways. This model further predicts the existence of distinct signaling complexes defined by the LRR domain conformations of FLS2 and BAK1, which are specifically assigned to diverse signaling outputs. Such complexes might be separated spatiotemporally from each other and/or different in the composition of accessory proteins.

## SUBCELLULAR PARTITIONING AND TURNOVER OF THE RECEPTOR IN PATTERN RECOGNITION RECEPTORS SIGNALING

The mechanisms underlying the subcellular partitioning of PRRs between their biogenesis and functional sites are still poorly understood in plants (Popescu, 2012). The reticulon-like proteins RTNLB1/RTNLB2 regulate the accumulation of functional FLS2 at the PM, possibly by controlling the ER exit of FLS2 (Lee et al., 2011). In rice, a chaperone complex consisting of Hsp90 and its co-chaperon Hop/Sti1 promote the delivery of CERK1 from the ER to the PM (Chen et al., 2010). This work also raises the possibility that PRRs associate with their signaling partners during their travel in the secretory pathway. Detailed cell biological studies are needed to precisely decipher the subcellular dynamics of these immune receptors.

PRR ubiquitination has emerged as a determinant for the receptor levels by targeting the receptors for degradation and/or modulating their membrane trafficking. Direct ubiquitination and subsequent degradation of the receptor contributes to signal attenuation for FLS2 (Lu et al., 2011). Upon flg22 binding, the FLS2 complex recruits two related U-box E3 ligases, PUB12 and PUB13, along with BAK1. A flg22-induced increase in BAK1 kinase activity on PUB12/PUB13 suggests a model in which BAK1-mediated phosphorylation promotes PUB12/13-mediated FLS2 ubiquitination. However, given that SOBIR1 is required to stabilize Cf-4 (Liebrand et al., 2013b), degradation of one of the receptor-interacting proteins might cause the receptor destabilization. Future investigation is required for how trans-phosphorylation between FLS2 and BAK1 influences the recruitment and phosphorylation of PUB12/PUB13 and how PUB phosphorylation influences the E3 ligase activity. Another E3 ligase triplet, PUB22/23/24 also acts as a negative regulator for FLS2, EFR, and PEPR signaling (Trujillo et al., 2008; Stegmann et al., 2012). Yeast two-hybrid screens for the E3 ligase interactors led to the identification of the exocyst subunit EXO70B2 as a target for PUB22-mediated ubiquitination (Stegmann et al., 2012). EXO70B2 contributes to different PTI-associated outputs, implying its influence on the receptor function, possibly through maintaining the PM receptor pool. The defects of *exo70b2* mutants in responses to different MAMPs and pathogens point to a role for EXO70B2 in a common step between different receptor pathways. Exocyst subunit degradation might lower the delivery of these receptors to the PM. However, compared to the *pub *triple mutants, the defects of *exo70b2* mutants in PTI-related outputs are much smaller, implying the existence of another ubquitination target(s) for these E3 ligases. Interestingly, the *Medicago truncatula *E3 ligase PUB1 has been also described to interact with and negatively regulate the LysM-RLK LYK3-mediated nodulation (Mbengue et al., 2010). This molecular logic might be widespread for transmembrane receptors in plant-microbe interactions.

Pattern recognition receptors undergo ligand-induced internalization from the PM, as described for FLS2 and LeEIX2 (Robatzek et al., 2006; Bar and Avni, 2009). Although this process is closely correlated with immune signaling activation, it remains to be determined whether it serves signal activation or attenuation of the PRRs. Recent studies showed that flg22 perception transiently lowers the steady-state FLS2 levels and causes signal de-sensitization, which is followed by the replenishment of FLS2 accumulation and signal re-sensitization (Smith et al., 2013). It is of high interest to correlate this turnover event with subcellular dynamics of the receptor.

## ENDOPLASMIC RETICULUM STRESS AND DEFENSE REGULATION

Overloading of misfolded proteins beyond the capacity of ERQC, termed ER stress, induces the so-called unfolded protein response (UPR) that is characterized by the induction of ER chaperones. Although close associations between UPR, disease, and immunity have been well documented in animals (Todd et al., 2008), much less information is available in plants (Vitale and Boston, 2008; Eichmann and Schafer, 2012). Two arms of UPR signaling have been described in *Arabidopsis* that involve the transmembrane transcription factors (TFs) bZIP17/bZIP28 and the protein kinase/ribonuclease IRE1 (Iwata and Koizumi, 2012; Howell, 2013). IRE1 serves to generate an active form of the TF bZIP60 via its mRNA processing. The two homologues IRE1a and IRE1b, as well as bZIP60, contribute to SA-mediated antibacterial immunity (Moreno et al., 2012). It seems likely that UPR-based increase of ERQC capacity ensures the supply of functional transmembrane regulators and alleviates cell death or damages that are caused by excessive ER stress (Howell, 2013). Indeed, *ire1a ire1b* plants show enhanced cell death upon ER stress, pointing to a role for the proper UPR in the ER homeostasis (Mishiba et al., 2013). ER stress-induced cell death is exploited by the mutualistic fungus *Piriformospora indica* for *Arabidopsis* root infection (Qiang et al., 2012). The fungal colonization is dependent on active suppression of the host UPR, ER disintegration and subsequent vacuolar processing enzyme-mediated vacuolar collapse leading to cell death in the colonized cells. Impaired ER integrity might disturb vesicular secretion of antimicrobial and/or defense signaling molecules, which could also facilitate fungal colonization.

Excessive ER stress also induces autophagy, another link to plant defense responses (see more details in Hayward and Dinesh-Kumar, 2011; Teh and Hofius, 2014). Both pro-survival and pro-death functions have been assigned to autophagy in the control of immune responses. Likewise, excessive ER stress can also positively influence plant immunity. In this respect, it is conceivable that ER stress-induced cell death emits DAMPs, as documented in animals (Krysko et al., 2013). This possibility remains understudied in plants. ER stress induces the TF OsWRKY45 that promotes SA-based defense in rice (Hayashi et al., 2012) and systemic immunity in cucumber (Sticher and Metraux, 2000). The mechanisms by which excessive ER stress is sensed and linked to defense activation represent an important future challenge.

## CONCLUSION AND PERSPECTIVE

The ER regulates the abundance, quality and signaling function of transmembrane immune receptors. Genetic and proteomic studies led to the identification of ERQC components that are critical for PRR biogenesis in different plant species. Interestingly, emerging evidence points to a role for ERQC in receptor signal sorting, possibly through controlling the folding states and thus conformations of the extracellular domains. Future studies will be needed to reveal how ERQC modulates the turnover, subcellular dynamics, complex assembly, and post-recognition signaling of PRRs. It is also of importance to show how PRR biogenesis is rewired during pathogen challenges, according to the extent of ER stress arisen. Excessive ER stress might facilitate to engage DAMP-mediated immune systems that are tolerant to ERQC dysfunction, and thereby can ultimately enhance plant immunity.

## Conflict of Interest Statement

The authors declare that the research was conducted in the absence of any commercial or financial relationships that could be construed as a potential conflict of interest.
